# Cutaneous leishmaniasis due to *Leishmania (L.) mexicana*: first imported case reported in Portugal^؆^

**DOI:** 10.1016/j.abd.2025.501223

**Published:** 2025-10-27

**Authors:** Mélissa M. de Carvalho, Sofia Cortes, José Manuel Cristóvão, José Carlos Cardoso, Maria Goreti Catorze, Alexandre Miroux-Catarino

**Affiliations:** a Dermatovenereology Service, Hospital de Egas Moniz, Unidade Local de Saúde de Lisboa Ocidental, Lisbon, Portugal; b Associated Laboratory in Translation and Innovation Towards Global Health REAL, Global Health and Tropical Medicine Research and Development Center, Institute of Hygiene and Tropical Medicine, NOVA University of Lisbon, Lisbon, Portugal; c Dermatovenereology Service, Unidade Local de Saúde de Coimbra, Coimbra, Portugal

Dear Editor,

Leishmaniasis is a parasitic disease caused by protozoa of the genus *Leishmania*, transmitted through the bite of infected phlebotomine sandflies.^1^, ^2^, ^3^ Leishmaniasis is endemic in tropical, subtropical, and Mediterranean regions, with its distribution spanning the Old World (Africa, Asia, the Middle East, the Mediterranean Basin) and the New World (Central and South America).^3^ Leishmaniasis has different clinical presentations, including visceral leishmaniasis (VL), cutaneous leishmaniasis (CL) and mucocutaneous leishmaniasis (ML).^1^, ^3^, ^4^ In Portugal, VL is endemic, with dogs serving as the primary reservoir and CL is considered a rare disease.^5^, ^6^

CL typically presents in exposed areas (e.g. face, neck, extremities) as painless solitary or multiple papulonodules that may progress to necrosis, ulceration, and scarring.^1^, ^4^

*Leishmania (L.) mexicana* is primarily found in Central America, Mexico, and Texas.^4^ This species is associated with small, chronic cutaneous ulcers, with a spontaneous healing rate exceeding 75% within three months.^1^, ^4^

We report the case of a 32-year-old man with an erythematous nodule on the chin, showing central ulceration covered by yellowish exudate and crusts, with a documented progression over three months (Fig. 1). Six months prior, he had travelled to Mexico (Bacalar jungle) and had been previously treated with antibiotics, without improvement. Initial wound cultures isolated *Klebsiella aerogenes*, prompting treatment with targeted ciprofloxacin for three weeks, with partial improvement. Histopathological examination revealed dermal macrophages containing numerous intracellular *Leishmania* amastigotes (Figure 2, Figure 3), further corroborated by a positive skin culture. *L. mexicana* was identified by molecular techniques using Internal Transcribed Spacer 1 - Polymerase Chain Reaction - Restriction Fragment Length Polymorphism (ITS1-PCR-RFLP) and Heat Shock Protein 70 (HSP70) sequencing.^7^ The patient was treated with itraconazole (200 mg every 12 hours) for three months, with complete resolution (Fig. 4). To the best of our knowledge, this is the first identification of CL caused by *L. mexicana* in Portugal, representing an imported infection. Imported cases of CL caused by *L. mexicana* complex species have been reported in Europe, though they remain rare.^8^

**Figure 1:**
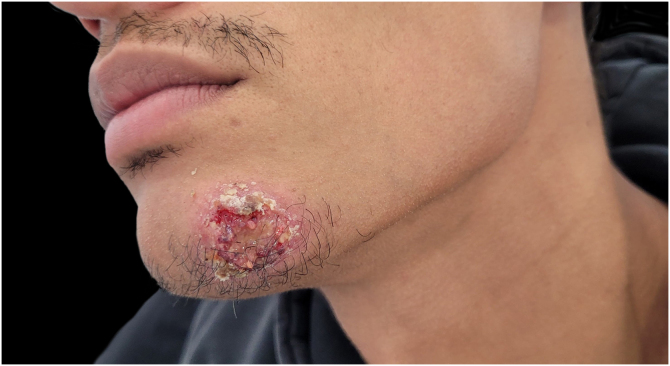
Physical examination revealing a 3 × 3 cm erythematous nodule on the chin, with central ulceration covered by yellowish exudate and crusts.

**Figure 2:**
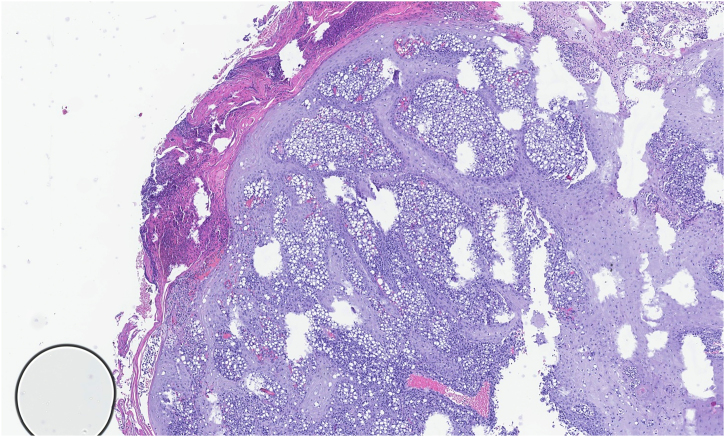
Histopathological examination showing a cutaneous nodule with epidermal hyperplasia and a dense dermal infiltrate extending into the deep dermis and subcutaneous tissue, with areas of granulomatous inflammation (Hematoxylin & eosin, ×10).

**Figure 3:**
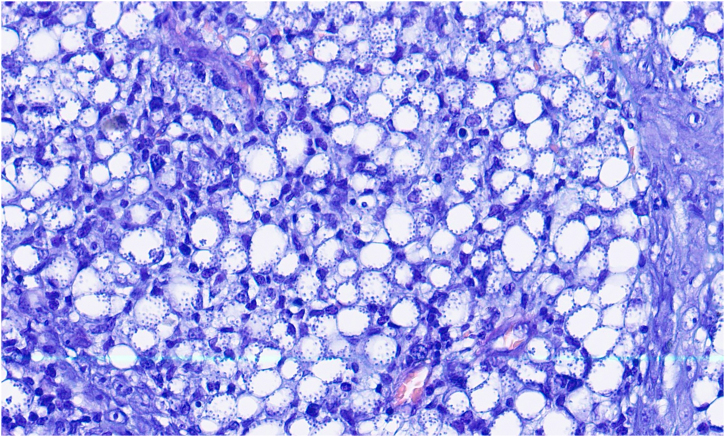
Histopathological examination showing dermal macrophages containing numerous intracellular amastigotes (Giemsa, ×1000).

**Figure 4:**
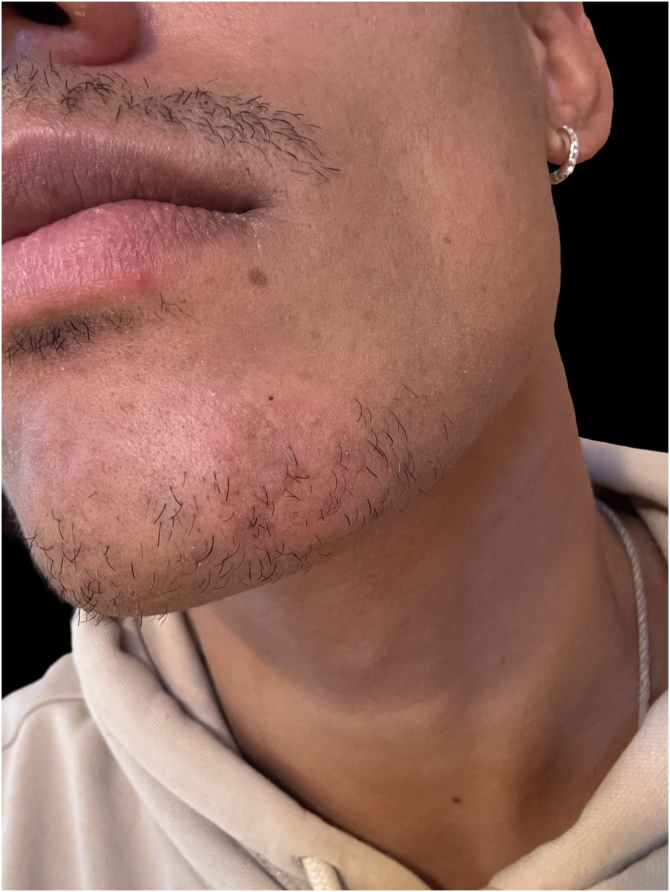
Physical examination reveals only post-inflammatory hyperpigmentation.

CL should be suspected in patients with cutaneous non-healing lesions and a history of exposure to endemic areas.^1^, ^2^, ^4^ Differential diagnoses include other infectious conditions (e.g., ecthyma, atypical mycobacterial infections, deep fungal infections), pyoderma gangrenosum, and neoplasms.^1^, ^4^ Accurate diagnosis requires a high index of suspicion and the integration of clinical, histopathological, and molecular findings. Our case highlights the importance of Dermatologists remaining vigilant for imported cases of CL.^9^ The growing number of imported diseases in Europe, such as CL, reflects the impact of globalization, driven by increased international travel and migration. While the likelihood of exotic *Leishmania* species establishing in non-endemic areas is relatively low, due to the absence of their primary reservoir hosts, the potential for local sand fly species to act as competent vectors cannot be disregarded. This raises the possibility that non-native *Leishmania* species could adapt and spread, leading to notable public health concerns.^10^

In cases of complex lesions (e.g. facial lesions), systemic treatment is warranted. First-line systemic therapies include meglumine antimoniate or liposomal amphotericin B.^1^, ^9^ Recent guidelines support oral miltefosine as a convenient, effective alternative. Imidazoles may also be considered for theirconvenience and security, though prolonged courses and high doses may lead to adverse effects (e.g. hepatotoxicity).^9^ Treatment outcomes are monitored based on clinical resolution, including re-epithelialization, regression of infiltration and erythema, and lesion flattening, with follow-up recommended for up to 12 months post-treatment.^1^

Bacterial superinfection, as observed in this case, is an important complication of CL. The presence of *Klebsiella aerogenes* likely contributed to the lesion’s size, and exudation, and a delayed diagnosis. While this organism is rarely a colonizer, its pathogenic role was further supported by the lesion’s initial improvement with targeted antibiotic therapy.

This report documents the first identified case of *L. mexicana* in Portugal, representing an imported infection from Mexico. It emphasizes the need for awareness of CL as a differential diagnosis for cutaneous ulcers, particularly in patients with travel histories to endemic regions. Prompt diagnosis and timely treatment are essential to prevent complications. This case further highlights the importance in recognizing the role of bacterial superinfection and highlights itraconazole as an effective treatment option. Continued surveillance and awareness are essential to manage the growing incidence of imported diseases in dermatological practice.

## ORCID IDs

Sofia Cortes: 0000-0001-5850-6950

José Manuel Cristóvão: 0000-0003-3861-9346

José Carlos Cardoso: 0000-0001-7113-7061

Maria Goreti Catorze: 0000-0003-2633-1249

Alexandre Miroux-Catarino: 0000-0001-8243-8724

## Financial support

None declared.

## Authors’ contributions

Mélissa M. de Carvalho: Conception and study design; drafting the manuscript; critical review of the literature.

Sofia Cortes: Data collection; drafting the manuscript and critical intellectual review; acquisition and interpretation of laboratory data.

José Manuel Cristóvão: Data collection; critical intellectual review; acquisition and interpretation of laboratory data.

José Carlos Cardoso: Critical intellectual review; acquisition and interpretation of histological data and iconographic records; intellectual participation in the clinical management of studied cases.

Maria Goreti Catorze: Critical intellectual review.

Alexandre Miroux-Catarino: Critical intellectual review; supervision of the research; acquisition and interpretation of iconographic records.

## Research data availability

Does not apply.

## Conflicts of interest

None declared.
